# TEG®- or ROTEM®-based individualized goal-directed coagulation algorithms: don’t wait - act now!

**DOI:** 10.1186/s13054-014-0637-3

**Published:** 2014-11-24

**Authors:** Donat R Spahn

**Affiliations:** Institute of Anesthesiology and Head Medical, Section Anesthesiology, Intensive Care Medicine and OR-Management, University and University Hospital Zurich, 8091 Zurich, Switzerland

## Abstract

In trauma patients, TEG® and ROTEM® allow prediction of massive transfusion requirement and mortality, and creation of goal-directed, individualized coagulation algorithms that may improve patient outcome. This outcome benefit has been shown for cardiac surgery in prospective randomized trials. For trauma, only non-randomized studies have been performed. Nevertheless, TEG® and ROTEM® are highly promising monitoring techniques to guide coagulation management in all types of major bleeding, including trauma.

The review by Da Luz and colleagues in this issue of *Critical Care* highlights the progress provided by thrombelastography (TEG®) and thrombelastometry (ROTEM®) in diagnosing and monitoring the coagulation system in trauma patients [1]. Their systematic review includes 55 studies and over 12,000 patients and they find that early abnormalities in TEG®/ROTEM® predict massive transfusion need and mortality. However, they conclude that 'Effects on blood product transfusion, mortality and other patient-important outcomes remain unproven in randomized trials' [1]. Formalistically this is of course correct; however, which monitoring system or laboratory value in medicine has ever improved relevant patient outcomes such as length of ICU or hospital stay, complications, treatment costs or even mortality? The answer is none, simply because a monitoring system or a laboratory value provides information allowing risk assessment but lacks any therapeutic potential.

TEG®/ROTEM® information, however, allows creation of goal-directed, individualized treatment algorithms that may improve patient outcome. This has been shown for TEG®/ROTEM®-based algorithms in prospective randomized studies in cardiac surgery [2,3]. In liver transplantation such algorithms have even become standard. And the benefits are impressive: reduced transfusion needs, less complications, shorter length of ICU and hospital stay, better survival and reduced treatment costs [2,3]. TEG®/ROTEM®-based algorithms have also been successful in improving patient outcome in trauma, although these studies were not prospective and randomized [4-6]. Nevertheless, the recommendation to use TEG®/ROTEM® in the treatment of severely injured trauma patients was upgraded in the 2013 European Trauma Treatment Guidelines from 2C to 1C with a plea to implement goal-directed, individualized treatment algorithms and to monitor treatment adherence [4].

TEG®, and even more so ROTEM®, point to the most critical element of coagulation within approximately 5 to 10 minutes [7] (compared with traditional laboratory analyses with turnaround times of consistently 60 minutes or more [1]), and allow diagnosis of even mild forms of hyperfibrinolysis that are not detectable by standard laboratory tests but are associated with increased mortality [8]. In the modern emergency room the first blood from a severely injured patient thus goes immediately into a ROTEM® device, the blood gas analyser, the central laboratory and the blood bank, and 1 gram of tranexamic acid is administered immediately thereafter. This provides within 10 minutes a baseline analysis of the coagulation situation and allows goal-directed specific treatment of the most critical deficit with coagulation factor concentrates [4-6]. The ROTEM® analysis is repeated again soon after to assess treatment success and to capture the dynamic evolution of the coagulation situation. This concept allows the patient to be treated sufficiently with the lowest dose of coagulation factors aiming at low normal coagulability and avoiding hypercoagulability. This is important for limiting treatment costs as well as avoiding thrombotic complications.

The success of the above concept has been shown in many studies [1-6,9], although a 'perfectly' designed prospective randomized double-blind multicentre study has not yet been performed to formalistically 'prove' its superiority. Is this a problem? My personal answer is: maybe. Sure, it would be nice to have such a study that would satisfy experts on a theoretical level. However, having served on numerous study-design committees of such studies, I have to admit that the ideal study design is extremely difficult to find. One crucial question is the definition of the control group. Is the control group also treated according to an individualized goal-directed algorithm or simply by a 1:1(:1) (red blood cell:plasma(:platelet)) transfusion regimen? Does the control group receive only labile blood products or also factor concentrates to treat coagulation abnormalities if present? The choice between a simple 1:1:1 transfusion regimen versus any individualized goal-directed algorithm has become particularly difficult after the recent prospective randomized study showing a more than two-fold increased mortality (32%) in the 1:1:1 transfusion regimen compared with a traditional laboratory-based individualized goal-directed treatment algorithm (14%) [9].

In addition, even if such a 'perfect' study were to be performed, its interpretation would be extremely difficult. In the case of lack of a significant outcome difference, the discussion would be that too few patients were included and thus the study would have been underpowered or the two treatment regimens were not sufficiently different from one another. In the case of a significant difference, the interpretation would be even more difficult and controversial: is the difference due to a different delay in the specific treatment of coagulopathy, or the fact that in one of the arms coagulation factors were used whereas in the other more blood products were used, or very generally because one of the algorithms was not good enough to provide a good outcome. This is by no means to say that we should stop doing outcomes research on coagulation management in severely injured patients, but that we should not dismiss existing evidence in favour of TEG®/ROTEM®-based goal-directed individualized coagulation algorithms on the basis that we lack the ultimate 'perfect' study. As a matter of fact, today all hospitals should have an individualized and goal-directed coagulation algorithm [4]: don’t wait - act now!

## References

[1] Da Luz LT, Nascimento B, Shankarakutty AK, Rizoli S, Adhikari NKJ (2014). Effect of thromboelastography (TEG®) and rotational thromboelastometry (ROTEM®) on diagnosis of coagulopathy, transfusion guidance and mortality in trauma: descriptive systematic review. Crit Care.

[2] Weber CF, Gorlinger K, Meininger D, Herrmann E, Bingold T, Moritz A, Cohn LH, Zacharowski K (2012). Point-of-care testing: a prospective, randomized clinical trial of efficacy in coagulopathic cardiac surgery patients. Anesthesiology.

[3] Rahe-Meyer N, Solomon C, Hanke A, Schmidt DS, Knoerzer D, Hochleitner G, Sorensen B, Hagl C, Pichlmaier M (2013). Effects of fibrinogen concentrate as first-line therapy during major aortic replacement surgery: a randomized, placebo-controlled trial. Anesthesiology.

[4] Spahn DR, Bouillon B, Cerny V, Coats TJ, Duranteau J, Fernandez-Mondejar E, Filipescu D, Hunt BJ, Komadina R, Nardi G, Neugebauer E, Ozier Y, Riddez L, Schultz A, Vincent JL, Rossaint R (2013). Management of bleeding and coagulopathy following major trauma: an updated European guideline. Crit Care.

[5] Schochl H, Nienaber U, Maegele M, Hochleitner G, Primavesi F, Steitz B, Arndt C, Hanke A, Voelckel W, Solomon C (2011). Transfusion in trauma: thromboelastometry-guided coagulation factor concentrate-based therapy versus standard fresh frozen plasma-based therapy. Crit Care.

[6] Schoechl H, Nienaber U, Hofer G, Voelckel W, Jambor C, Scharbert G, Kozek-Langenecker S, Solomon C (2010). Goal-directed coagulation management of major trauma patients using rotation thromboelastometry (ROTEM)-guided administration of fibrinogen and prothrombin complex concentrate. Crit Care.

[7] Theusinger OM, Nurnberg J, Asmis LM, Seifert B, Spahn DR (2010). Rotation thromboelastometry (ROTEM) stability and reproducibility over time. Eur J Cardiothorac Surg.

[8] Chapman MP, Moore EE, Ramos CR, Ghasabyan A, Harr JN, Chin TL, Stringham JR, Sauaia A, Silliman CC, Banerjee A (2013). Fibrinolysis greater than 3% is the critical value for initiation of antifibrinolytic therapy. J Trauma Acute Care Surg.

[9] Nascimento B, Callum J, Tien H, Rubenfeld G, Pinto R, Lin Y, Rizoli S (2013). Effect of a fixed-ratio (1:1:1) transfusion protocol versus laboratory-results-guided transfusion in patients with severe trauma: a randomized feasibility trial. CMAJ.

